# Onset of pain to surgery time in acute aortic dissections type A: a mandatory factor for evaluating surgical results?

**DOI:** 10.3389/fcvm.2023.1102034

**Published:** 2023-04-27

**Authors:** Tim Kaufeld, Andreas Martens, Erik Beckmann, Linda Rudolph, Heike Krüger, Ruslan Natanov, Morsi Arar, Wilhelm Korte, Tobias Schilling, Axel Haverich, Malakh Shrestha

**Affiliations:** ^1^ Department of Cardiothoracic, Transplant and Vascular Surgery, Hannover Medical School, Hannover, Germany; ^2^ Minneapolis Heart Institute, Abbott Northwestern Hospital, Minneapolis, MN, United States; ^3^ Cardiovascular Medicine, Mayo Clinic, Rochester, MN, United States

**Keywords:** aortic dissection, AADA, arch repair, time, pain

## Abstract

**Objective:**

An acute aortic dissection type A (AADA) is a rare but life-threatening event. The mortality rate ranges between 18% to 28% and mortality is often within the first 24 h and up to 1%–2% per hour. Although the onset of pain to surgery time has not been a relevant factor in terms of research in the field of AADA, we hypothesize that a patient's preoperative conditions depend on the length of this time.

**Methods:**

Between January 2000 and January 2018, 430 patients received surgical treatment for acute aortic dissection DeBakey type I at our tertiary referral hospital. In 11 patients, the exact time point of initial onset of pain was retrospectively not detectable. Accordingly, a total of 419 patients were included in the study. The cohort was categorized into two groups: Group A with an onset of pain to surgery time < 6 h (*n* = 211) and Group B > 6 h (*n* = 208), respectively.

**Results:**

Median age was 63.5 years (y) ((IQR: 53.3–71.4 y); (67.5% male)). Preoperative conditions differed significantly between the cohorts. Differences were detected in terms of malperfusion (A: 39.3%; B: 23.6%; P: 0.001), neurological symptoms (A: 24.2%; B: 15.4%; P: 0.024), and the dissection of supra-aortic arteries (A: 25.1%; B: 16.8%; P: 0.037). In particular, cerebral malperfusion (A 15.2%: B: 8.2%; P: 0.026) and limb malperfusion (A: 18%, B: 10.1%; P: 0.020) were significantly increased in Group A. Furthermore, Group A showed a decreased median survival time (A: 1,359.0 d; B: 2,247.5 d; P: 0.001), extended ventilation time (A: 53.0 h; B: 44.0 h; P: 0.249) and higher 30-day mortality rate (A: 25.1%; B: 17.3%; P: 0.051).

**Conclusions:**

Patients with a short onset of pain to surgery time in cases of AADA present themselves not only with more severe preoperative symptoms but are also the more compromised cohort. Despite early presentation and emergency aortic repair, these patients show increased chances of early mortality. The “onset of pain to surgery time” should become a mandatory factor when making comparable surgical evaluations in the field of AADA.

## Introduction

An acute aortic dissection type A is a rare but life-threatening event (1, 2). This severe disease can have various manifestations. Following an initial intimal tear, blood is able to penetrate from the aortic lumen into the media layer, resulting in a separation of the aortic wall layers and the subsequent formation of a true and a false lumen. This process may result in either organ malperfusion and impairment or even in an aortic rupture. Acute aortic dissections Stanford type A (AADA) are reported with high perioperative mortality ranging from 18% to 28% and mortality within the first 24 h and up to 1%–2% per hour (3–6). Harris et al. found that a delay in time from diagnosis to surgery may be associated with a history of previous cardiac surgery, presentation without abrupt or any pain, and initial presentation to a nontertiary care hospital (7). However, results from the United Kingdom suggest that both short-term and long-term outcomes are significantly related to surgeons' experience (8).

Multiple publications present surgical outcomes following aortic repair in AADA. Nevertheless, is it essential to integrate the onset of pain to surgery time to evaluate and compare patients' preoperative conditions and, furthermore, to avoid selection bias? Existing studies calculate the time between onset and operation in days (9). Our department calculates it in hours.

It is common sense that a delayed diagnosis of aortic dissection will result in an increased mortality rate (10).

In our retrospective study we evaluated patients who received open aortic repair due to AADA in our department. We distinguished this cohort into two groups: one group under and one group above our median onset of pain to surgery time of 6 h.

We hypothesize that onset of pain to surgery time is a highly underrated factor in comparative evaluations regarding survival after aortic repair. Furthermore, we assume that a patient's preoperative conditions depend on this duration of time. But how should it be interpreted? The earlier the better?

## Methods

### Study population and study design

Between January 2000 and January 2018, 430 patients received surgical treatment due to acute aortic dissection DeBakey type I at our tertiary referral hospital. In 11 patients, the exact time point of initial onset of pain was retrospectively not detectable. Accordingly, a total of 419 patients (67.5% male; 63.5 years (y) median age; interquartile range (IQR) 53.3–71.4 y) were included in our study. All 430 patients were comers. Iatrogenic dissection and AADAs that occurred inside our hospital were not included in this study to prevent further selection bias.

DeBakey type II and III and chronic dissections were not included in this study. Due to the median onset of pain to surgery time of 6 h (h), we categorized the cohort in two groups: Group A with an onset of pain to surgery time < 6 h (*n* = 211) and Group B > 6 h (*n* = 208), respectively. Data were collected at our aortic outpatient clinic where patients were frequently seen after surgery. Furthermore, patients were actively contacted by a study nurse team. Individual consent was obtained from all patients to allow for further follow-up data collection. Follow-up data collection was ended and completed in February 2022. Our retrospective study was approved by our institutional ethics committee. Patients' preoperative characteristics are presented in Tables 1, 2.

**Table 1:** Patients’ characteristics: IQR, interquartile range; BMI, body mass index; PVOD, peripheral vascular occlusion disease; COPD, chronic obstructive pulmonary disease.

Characteristics	Entire cohort	Onset of pain </=6 h	Onset of pain > 6 h	*P*-value
Total patients	*n* = 419	*n* = 211	*n* = 208	
Age at surgery (years), median (IQR)	63.5 (53.3–71.4)	63.7 (53.1–71.5)	63.0 (53.6–71.1)	0.656
Sex male, *n* (%)	283 (67.5)	149 (70.6)	134 (64.4)	0.176
BMI, median (IQR)	26.2 (24.2–29.1)	26.1 (24.2–29.2)	26.3 (24.0–28.1)	0.574
Hypertension, *n* (%)	270 (64.4)	131 (62.1)	139 (66.8)	0.311
Diabetesmellitus,*n*(%)	29 (6.9)	15 (7.1)	14 (6.7)	0.879
PVOD, *n* (%)	18 (4.3)	7 (3.3)	11 (5.3)	0.320
COPD, *n* (%)	39 (9.3)	15 (7.1)	24 (11.5)	0.119
Coronary heart disease, *n*(%)	42 (10.0)	16 (7.6)	26 (12.5)	0.094
Hyperthyreosis, *n* (%)	3 (0.7)	1 (0.5)	2 (1.0)	0.621
Hypothyreosis, *n* (%)	33 (7.9)	15 (7.1)	18 (8.7)	0.557
Atrial fibrillation, *n*(%)	52 (12.4)	20 (9.5)	32 (15.4)	0.067
Marfan syndrome, *n*(%)	19 (4.5)	7 (3.3)	12 (5.8)	0.228
Pericardialtamponade,*n*(%)	162 (38.7)	91 (43.1)	71 (34.1)	0.059
Bicuspid aortic valve, *n*(%)	19 (4.5)	13 (6.2)	6 (2.9)	0.107
Preoperativeintubation,*n*(%)	54 (12.9)	29 (13.7)	25 (12.0)	0.598
Mechanicalresuscitation, *n* (%)	38 (9.1)	23 (10.9)	15 (7.2)	0.189
Cardiac reoperation, *n*(%)	14 (3.3)	5 (2.4)	9 (4.3)	0.265

**Table 2:** Preoperative data: CT, computed tomography; LCA, left coronary artery; RCA, right coronary artery; IQR, interquartile range.

Characteristics	Entire cohort	Onset of pain </=6 h	Onset of pain > 6 h	*P*-value
Malperfusion, *n* (%)	132 (31.5)	83 (39.3)	49 (23.6)	**0.001**
Cerebralmalperfusion,*n*(%)	49 (11.7)	32 (15.2)	17 (8.2)	**0.026**
Visceral malperfusion, *n* (%)	35 (8.4)	22 (10.4)	13 (6.3)	0.122
Renal malperfusion, *n* (%)	47 (11.2)	29 (13.7)	18 (8.7)	0.099
Limb malperfusion, *n* (%)	59 (14.1)	38 (18.0)	21 (10.1)	**0.020**
Hemiparesis, *n* (%)	26 (6.2)	13 (6.2)	13 (6.3)	0.970
Paraparesis, *n* (%)	15 (3.6)	10 (4.7)	5 (2.4)	0.198
Seizure, *n* (%)	6 (1.4)	3 (1.4)	3 (1.4)	1.000
Evidence of stroke CT, *n* (%)	26 (6.2)	16 (7.6)	10 (4.8)	0.239
Neurologic symptoms, *n* (%)	83 (19.8)	51 (24.2)	32 (15.4)	**0.024**
Dissection supra-aortic arteries, *n* (%)	88 (21.0)	53 (25.1)	35 (16.8)	**0.037**
Dissection LCA, *n* (%)	12 (2.9)	6 (2.8)	6 (2.9)	0.980
Dissection RCA, *n* (%)	41 (9.8)	26 (12.3)	15 (7.2)	0.078
Onset of pain prior to surgery (h), median (IQR)	6.0 (4.0–13.0)	4.0 (3.0–5.0)	13.0 (8.0–30.0)	**<.001**

### Definitions

Patients with AADA may either present specific symptoms like floating back pain, abdominal pain, neurological disabilities, signs of malperfusion, or unspecific symptoms. CT angiography remains the gold standard for diagnosis of this potentially lethal disease. The existence of a dissection membrane starting in the ascending aorta or even an intramural hematoma inside the aortic wall represents the radiographic equivalent of an AADA. The onset of pain to surgery time was defined as the time from the documented painful event until skin cut in the operation theater.

Patients who presented themselves with severe neurologic symptoms like apraxia, hemiplegia, or dysarthria without a performed cerebral CT scan prior to surgery and postoperative evidence of stroke were assigned to the preoperative stroke cohort. A stroke had to be verified by CT or magnetic resonance imaging (MRI).

Occlusion or complete false lumen perfusion was defined as malperfusion according to Sievers et al. (11) (TEM Aortic Dissection Classification stage M2 and M3 ((−), (+)). The diagnosis of dissection of the coronary arteries was detected either using coronary angiography or had to be visible intraoperatively. AADAs accidently induced during open heart surgery were defined as iatrogenic dissection. Dissections postoperatively detected using CT or MRI were defined as *persisting dissections*.

For the diagnosis of hypertension, diabetes mellitus, or chronic obstructive pulmonary disease (COPD) a preoperatively performed medical treatment was necessary.

### Perioperative management and surgical technique

Our department provides emergency medical service to a population of approximately 2 million citizens. Longest distance of the ground- and helicopter-based patient transfer is around 100 km. According to our standard operating procedure, transfer to the operation theater must be performed promptly after diagnosis of AADA. Furthermore, we established a rapid response team of aortic surgeons, able to provide aortic repair 24/7 in case of AADA. These standardized procedures result in a median time from onset of pain to surgery of approximately 6 h.

According to our standard operating procedure, transfer to the operation theater must be performed promptly after diagnosis of AADA. To avoid early cardiac decompensation, intubation was not performed until all anesthesiological and surgical preparations were completed. This was followed by intubation and the establishment of full sternotomy extra-corporal circulation (ECC). Our cannulation technique in cases of AADA was previously published by our group (12, 13). We prefer direct aortic cannulation. Following the identification of the true lumen using transesophageal echocardiography, direct cannulation was performed. The left side of the heart was vented via the right upper pulmonary vein. The application of cardioplegia was performed directly into the coronary ostia and repeated every 30 min. Blood cardioplegia was used for myocardial protection. In 2010 we established the beating heart technique for extended arch surgery (14).

The root first technique was performed while cooling the patient. Concomitant procedures (e.g., CABG) were performed during cooling if necessary. In all cases of AADA with extended arch surgery, hypothermic circulatory arrest (temperatures between 22°C and 26°C) and bilateral selective antegrade cerebral perfusion were performed. The use of SACP varied based on the surgeon's decision on whether proximal arch repair was to be done.

### Extended arch repair

From 2000 to 2010, the FET technique was performed using a custom-made Chavan-Haverich prosthesis followed by a prefabricated Chavan-Haverich hybrid graft (15, 16) (Curative GmbH, Dresden, Germany). From 2005 to 2010, the Jotec E-vita hybrid graft was used (17). The attachment of the supra-aortic arteries was performed using the island (*en bloc*) technique until 2010. Following the island technique, we switched to the four-branched frozen elephant trunk prosthesis (FET Vascutek Terumo, Terumo®, Glasgow, UK) (18, 19). We changed our strategy in 2007 from a straight graft with island technique to the branched Sienna™ graft (Terumo®, Glasgow, UK), even for total or hemi-arch replacement. The extensive use of branched aortic arch prostheses resulted in major technical developments. As a consequence of these changes, arch replacement was performed after completing cardiac and proximal aortic repair. Head vessels were anastomosed to the corresponding side branches of the graft at the end of the procedure.

### Proximal arch repair

Limited aortic repair in terms of a proximal arch replacement was performed using established straight Dacron grafts.

### Statistical analysis

SPSS 27 Statistics software (IBM Corp. released 2020; IBM SPSS Statistics for Windows, Version 27.0; Armonk, NY: IBM Corp.) was used for data analysis. A normal distribution of variables was calculated using the Kolmogorov–Smirnov test. Categorical variables are stated as absolute numbers (*n*) and proportions. Normally distributed continuous variables are stated as mean ± standard deviation, while continuous variables without normal distribution are stated as median and interquartile range (IQR). Fisher's exact test was used to detect differences in categorical variables. Differences in continuous variables were tested using the Mann-Whitney U test. Kaplan–Meier analysis and log rank were used for the evaluation of survival, and the log rank test was used to test for differences. We did not correct for multiple testing.

## Results

### Preoperative patient characteristics

Patient demographics are found in Table 1. Median age did not differ significantly between the groups (A: 63.7 y; B: 63.0 y; P:0.656). The majority were male patients (A: 70.6%; B: 64.4%; P: 0.176). Hypertension was a dominant diagnosis in the patients’ history (A: 62.1%; B: 66.8%; P: 0.311). Overall, medical history, including diabetes mellitus (A: 7.1%; B: 6.7%; P: 0.879) and PVOD (A: 3.3%; B: 5.3%; P: 0.320) were comparable in both cohorts. Group B showed higher COPD (A: 7.1%; B: 11.5%; P:0.119), coronary heart disease (A: 7.6%; B: 12.5%; P: 0.094), and atrial fibrillation (A: 9.5%; B: 15.4%; P: 0.067). In contrast, pericardial tamponade (A: 43.1%; B: 34.1%; P: 0.059) and mechanical resuscitation (A: 10.9%; B: 7.2%; P: 0.189) occurred slightly but not significantly more often in patients with a painful event to surgery time <6 h.

Significant differences were detected in terms of malperfusion (A: 39.3%; B: 23.6%; P: 0.001), neurological symptoms (A: 24.2%; B: 15.4%; P: 0.024) and the dissection of supra-aortic arteries (A:25.1%; B: 16.8%; P: 0.037). In particular, cerebral malperfusion (A: 15.2%; B: 8.2%; P: 0.026) and limb malperfusion (A: 18%; B: 10.1%; P: 0.020) were significantly increased in Group A. In addition, the incidence of isolated dissection of the right coronary artery was higher in this cohort (A: 12.3%; B: 7.2%; P: 0.078).

### Intraoperative data

Intraoperative data are shown in Table 3. Operation time (A:333.0 min; B: 323.0 min; P: 0.509), cardiopulmonary bypass time (A: 220.0; B: 213.0; P: 0.342), and aortic cross-clamp time (A: 124.0 min.; B: 323 min; P: 0.509) did not differ significantly between the two cohorts. Group A showed a higher demand for platelet concentrates (A: *n* = 3.0 (IQR: 2.0–4.0); B: *n* = 2.0 (IQR: 2.0–4.0); P: 0.011). The number of operations extended in terms of limited vs. total arch repair was comparable in both groups. Neither limited nor extended aortic arch repair showed significant differences. In keeping with an elevated dissection rate of RCA, CABG was more often performed in Group A (A:19.9%; B: 14.9%; P: 0.177).

**Table 3:** Intraoperative data: SD, standard deviation; IQR, interquartile range; min, minute; HCA, hypothermic circulatory arrest time; CABG, coronary artery bypass graft; SACP, selective antegrade cerebral perfusion time.

Characteristics	Entire cohort	Onset of pain </=6 h	Onset of pain > 6 h	*P*-value
Total patients	*n* = 419	*n* = 211	*n* = 208	
Total operation time (min), median (IQR)	329.0 (259.0–405.0)	333.0 (259.0–411.0)	323.0 (257.0–387.8)	0.509
Cardiopulmonary bypass time (min), median (IQR)	217.0 (168.0–286.0)	220.0 (168.0–289.0)	213.0 (168.0–281.0)	0.342
Aortic cross-clamp time (min), median (IQR)	126.0 (93.0–162.0)	124.0 (92.0–162.0)	128.5 (94.0–161.8)	0.947
HCA (hypothermic circulatory arrest) time (min), median (IQR)	36.0 (25.0–52.0)	36.0 (25.0–51.0)	36.0 (24.3–55.0)	0.686
SACP (selective antegrade cerebral perfusion) time (min), median (IQR)	33.0 (20.0–76.0)	32.0 (19.0–83.0)	38.0 (20.0–72.0)	0.615
Minimal core temperature (°C), median (IQR)	24.6 (22.2–26.0)	24.5 (q22.0–26.0)	24.6 (22.3–26.0)	0.528
Erythrocyte concentrates, median (IQR)	6.0 (4.0–10.0)	6.0 (4.0–10.0)	6.0 (3.3–10.0)	0.884
Fresh frozen plasma, median (IQR)	6.0 (4.0–10.0)	6.0 (4.0–10.0)	6.0 (4.0–10.0)	0.685
Platelet concentrates, median (IQR)	3.0 (2.0–4.0)	3.0 (2.0–4.0)	2.0 (2.0–4.0)	**0.011**
**Arch replacement:**				
Proximal arch replacement, *n* (%)	186 (44.4)	96 (45.5)	90 (43.3)	0.646
Subtotal arch replacement, *n* (%)	34 (8.1)	15 (7.1)	19 (9.1)	0.448
Total arch replacement,*n* (%)	35 (8.4)	18 (8.5)	17 (8.2)	0.895
Total arch replacement elephant trunk, *n* (%)	46 (11.0)	17 (8.1)	29 (13.9)	0.054
Total Arch replacement frozen elephant trunk, *n*(%)	118 (28.2)	65 (30.8)	53 (25.5)	0.226
Bio glue, *n* (%)	141 (33.7)	78 (37.0)	63 (30.3)	0.148
**Aortic valve replacement:**				
Biologic, *n* (%)	62 (14.8)	30 (14.2)	32 (15.4)	0.737
Mechanic, *n* (%)	66 (15.8)	32 (15.2)	34 (16.3)	0.740
Rootinvolvement, *n*(%)	254 (60.6)	122 (57.8)	132 (63.5)	0.237
Bentall, *n* (%)	127 (30.3)	61 (28.9)	66 (31.7)	0.530
David, *n* (%)	97 (23.3)	49 (23.2)	48 (23.1)	0.972
Yacoub, *n* (%)	19 (4.5)	8 (3.8)	11 (5.3)	0.462
CABG, *n* (%)	73 (17.4)	42 (19.9)	31 (14.9)	0.177
ECMO, *n* (%)	18 (4.3)	9 (4.3)	9 (4.3)	0.975
Exitusintabula,*n*(%)	12 (2.9)	6 (2.8)	6 (2.9)	0.980

### Postoperative data

Patients with a shorter “onset of pain to surgery time” presented a significantly decreased median survival time (A:1,359.0 d; B: 2,247.5 d; P: 0.001), extended ventilation time (A: 53.0 h; B: 44.0 h; P: 0.249), and a higher 30-day mortality rate (A: 25.1%; B: 17.3%; P: 0.051). Furthermore, postoperative persisting visceral malperfusion (A: 4.7%; B: 0%; P: 0.002) and renal malperfusion (A: 8.1%; B: 1.4%; P: 0.001) occurred mostly in Group A (Table 4).

**Table 4:** Postoperative data: SD, standard deviation; IQR, interquartile range; min, minute; CCT, cranial computed tomography.

Characteristics	Entire cohort	Onset of pain </=6 h	Onset of pain > 6 h	*P*-value
Total patients	*n* = 419	*n* = 211	*n* = 208	
Survival time (days), median (IQR)	1,741.0 (80.0–3,223.0)	1,359.0 (24.0–2,786.0)	2,247.5 (309.5–3,740.0)	**0.001**
Ventilation time (h)	48.0 (21.0–136.0)	53.0 (23.0–146.0)	44.0 (20.0–110.8)	0.249
Intensive care unit (days), median (IQR)	4.0 (2.0–8.0)	4.0 (2.0–8.0)	4.0 (2.0–8.0)	0.784
Rethoracotomy,*n*(%)	69 (16.5)	37 (17.5)	32 (15.4)	0.553
Dialysis, *n* (%)	52 (12.4)	30 (14.2)	22 (10.6)	0.258
30-day mortality, *n*(%)	89 (21.2)	53 (25.1)	36 (17.3)	0.051
CCT stroke, *n* (%)	82 (19.6)	44 (20.9)	38 (18.3)	0.505
New-onset stroke,*n* (%)	35 (8.4)	18 (8.5)	17 (8.2)	0.895
Persisting cerebral malperfusion, *n*(%)	15 (3.6)	11 (5.2)	4 (1.9)	0.070
Persisting limb malperfusion, *n*(%)	11 (2.6)	5 (2.4)	6 (2.9)	0.742
Persisting visceral malperfusion, *n*(%)	10 (2.4)	10 (4.7)	0 (0.0)	**0.002**
Persisting renal malperfusion, *n*(%)	20 (4.8)	17 (8.1)	3 (1.4)	**0.001**

### Long-term follow-up data

**Figure 1:**
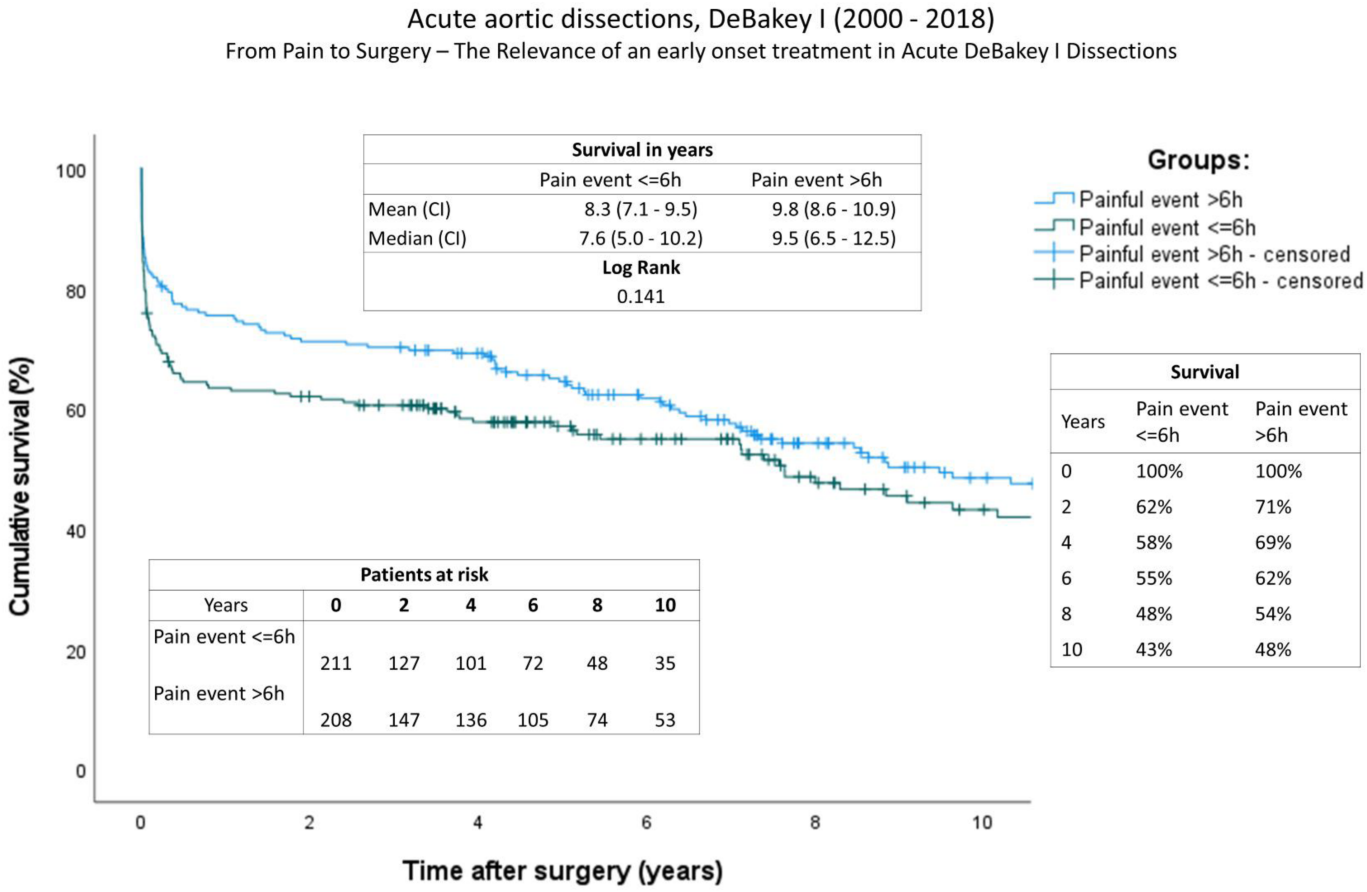
Survival: Kaplan-Meier curves showing survival of both groups (onset of pain <6 h; onset of pain >6 h). The x-axis denotes the time after operation.

The completeness of follow-up was 98,8% and the mean follow-up time for the entire group is 5.7 ± 5.4 years (2,079.7 ± 1,958.6 days). Follow-up data can be found in Table 5. The ratio of re-operations and re-interventions was comparable. A comparison of long-term survival using Kaplan-Meier curves is given in Figure 1. Significant differences were found between the groups. In contrast to a mean survival of 9.8 years (onset of pain > 6 h) (IQR: 8.6–10.9), survival time was decreased in patients with a short onset of pain to surgery time (<6 h) at 8.3 years (IQR: 7.1–9.5y), (log rank, P: 0.141). The survival rate at one year after aortic repair was A: 63% and B: 76%; at two years A: 62% and B: 71%; and A: 58% and B: 69% four years after surgery.

**Table 5:** Follow-up data: TAA, thoraco-abdominal aneurysm; TEVAR, thoracic endovascular aortic repair; EVAR, endovascular aneurysm repair.

Characteristics	Entire cohort	Onset of pain </=6 h	Onset of pain > 6 h	*P*-value
Total patients	*n* = 419	*n* = 211	*n* = 208	
Secondary aortic operation, *n* (%)	52 (12.4)	22 (10.4)	30 (14.4)	0.215
Re-operation identical area, *n* (%)	16 (3.8)	7 (3.3)	9 (4.3)	0.590
Re-operation downstream aorta, *n*(%)	36 (8.6)	15 (7.1)	21 (10.1)	0.275
TAA repair, *n* (%)	9 (2.1)	5 (2.4)	4 (1.9)	1,000
Y prosthesis, *n* (%)	4 (1.0)	0 (0.0)	4 (1.9)	0.060
Descending repair, *n*(%)	18 (4.3)	7 (3.3)	11 (5.3)	0.346
Hybrid, *n* (%)	7 (1.7)	4 (1.9)	3 (1.4)	1,000
TEVAR, *n* (%)	12 (2.9)	7 (3.3)	5 (2.4)	0.575
EVAR, *n* (%)	5 (1.2)	5 (2.4)	0 (0.0)	0.061
Aortic fenestration (%)	2 (0.5)	1 (0.5)	1 (0.5)	1.000

## Discussion

AADA is a catastrophic event characterized by a high mortality and a high urgency indication for surgical repair. Despite an extremely high early mortality rate of up to 35% in the first 24 h (20, 21), the duration from onset of pain to surgery has been a neglected topic in current AADA research. The comparability of early and long-term results requires reliable and almost identical preoperative conditions. Apart from the potentially high chance of selection bias due to the long time until aortic repair, we hypothesized that even a patient's preoperative status will differ significantly depending on the onset of pain to surgery time.

Nevertheless, an analysis from the International Registry of Acute Aortic Dissection (IRAD) indicates that the median time from emergency department presentation to a definitive diagnosis of acute aortic dissection is 4.3 h, with an additional 4 h between diagnosis and surgical intervention (22, 23). In comparison to IRAD data, in our department, patients with AADA received surgical treatment 6.0 h (median) following the initial painful event.

The time from “onset of pain” to surgery in acute aortic type A dissections depends on a variety of factors. Our department is well-established within the highly advanced German health care infrastructure. Clinical outcomes however depend on the diagnostic capacity of the referring center as well as on the speed of transportation and/or distance of travel. According to our data, patients' conditions from both cohorts varied substantially on admission to our hospital. Building on our thesis that strong clinical signs lead to a more rapid AADA diagnosis, Group A (<6 h) presented a higher incidence of independent risk factors for mortality, including pericardial tamponade (A: 43.1%; B: 34.1%; P: 0.059) and malperfusion (A: 39.3%; B: 23.6%; P: 0.001) (24). In particular, the increased incidence of cerebral malperfusion (A: 15.2%; B: 8.2%; P: 0.026) and limb malperfusion (A: 18.0%; B: 10.1%; P: 0.020) is consistent with more severe clinical symptoms. Furthermore, the high number of severe neurological symptoms (A: 24.2%; B: 15.4%; P: 0.024), which correlates with an increased incidence of the dissection of supra-aortic arteries (A: 25.1%; B: 16.8%; P: 0.037), is compatible with the thesis of early admission due to more severe symptoms.

There was no significant adjustment to the operative procedure observed in patients with aortic dissection <6 h. Even total operation time, cardiopulmonary bypass time, as well as aortic cross-clamp time, which are recognized as independent risk factors for mortality (25), were almost equally distributed in both groups.

Despite an equivalent surgical treatment, median survival time was significantly reduced (A: 1,359.0 d; B: 2,247.5 d; P: 0.001) and 30-day mortality (A: 25.1%; B: 17.3%; P: 0.051) increased in the cohort with the short onset of pain to surgery time (<6 h). This may be associated with the patient's assessed critically fatal conditions prior to surgery, with the consequence of a significantly reduced outcome despite comparable treatments.

These results correlate with a high rate of persisting visceral malperfusion (A: 4.7%; B: 0%; P: 0.002) and persisting renal malperfusion (A: 8.1%; B: 1.4%; P: 0.001) after surgery. According to the IRAD, malperfusion is the second most important cause of death after aortic dissection (20, 26, 27). There are two possible explanations for this remaining malperfusion. First, progress of the aortic dissection membrane of the downstream aorta between the initial CT scan and start of surgery, and second, the progress of the aortic dissection intraoperatively induced. Crawford et al. (27) previously described their observation that open aortic repair does not guarantee the restoration of distal perfusion as such, and end-organ malperfusion persists in up to 25% of patients, contributing to dismal operative outcomes (26). Nevertheless, only a minor number of malperfusions persist in patients with a longer time from pain onset to surgery. These results may imply an increased vulnerability of the aortic tissue during the early phase of AADA. To date, the existing literature needs to ask the relevant question of why patients with AADA show an elevated mortality rate during the first hours of disease occurrence, and whether an adjustment to their surgical therapy depending on the time factor is required. Until now, it was common sense that the early repair of aortic dissection would lead to a correction of malperfusion in most cases, while Goldberg et al. (28) suggested a better outcome when treating malperfusion first followed by a delayed repair of aortic dissection. The “malperfusion first” strategy is based on the fact that surgical repair can take a long time and persisting malperfusion may increase the chance of end-organ failure and aortic rupture (28). Finally, the question of whether an adjusted treatment is able to improve a patient's outcome or whether a patient's preoperative conditions are so limited that survival is independent of surgery remains unanswered.

Despite our results, the “early surgical treatment strategy” should remain the recommended algorithm in all cases to correct and prevent malperfusion or aortic rupture (29). However, whether this strategy includes the treatment of the more severely compromised patients should be clarified.

Nevertheless, our results also show that survival after an AADA depends on the health system infrastructure. Long transfer times due to geographic issues and prompt access to diagnostics and surgeon's skills are relevant factors for a patient's survival. It can be assumed that even in advanced healthcare systems, up to 20% of patients with AADA die before reaching a specialist center with surgical expertise for aortic surgery. Moreover, a diagnostic delay occurs in almost 40% of cases (30, 31).

According to our data, it cannot be denied that evidenced-based research in the field of AADA must consider the “onset of pain to surgery time” to avoid selection bias and to represent comparable results. Although this could be controversial, purely surgical results in terms of postoperative survival correlate positively with the onset of pain to surgery time: the longer the better.

## Limitations

This is a retrospective study, and thus carries potential risks and biases linked to studies of this nature. Furthermore, decisions about the surgical procedure were individually made by the surgeon. Between the years 2000–2018, a total of 21 surgeons at our center performed surgical aortic repair in cases of AADA. Surgical skill levels may vary in this cohort. There were relevant changes regarding the total arch approach during the observed period of time. The use of different of prosthesis and surgical techniques may influence the study result. Our results confirm an increasing mortality rate in patients with a short onset of pain to surgery time. Nevertheless, strategies for the adjustment of a corresponding therapy were not evaluated. The number of patients who died before reaching the hospital is not documented. Knowledge of the exact number of patients who died preoperatively could influence the impact of the study.

## Conclusion

Time is aorta. The prevention of high mortality due to AADA includes the development of a healthcare infrastructure for early diagnosis, referral, rapid hospital transfer, and well-trained aortic surgeons. In cases of AADA (where the onset of pain to surgery time is <6 h), patients present themselves not only with more severe preoperative symptoms but also are more compromised. Despite early presentation and emergency aortic repair, these patients show an increased chance of early mortality. The “onset of pain to surgery time” should become a required factor for making the surgical evaluation more comparable.

## Data Availability

The original contributions presented in the study are included in the article/Supplementary Material, further inquiries can be directed to the corresponding author/s.

## References

[1] Howard DP Banerjee A Fairhead JF Perkins J Silver LE Rothwell PM Population-based study of incidence and outcome of acute aortic dissection and premorbid risk factor control: 10-year results from the Oxford vascular study. Circulation. (2013) 127(20):2031–7. 10.1161/CIRCULATIONAHA.112.000483 23599348 PMC6016737

[2] Harris PD Bowman FO Jr Malm JR. The management of acute dissections of the thoracic aorta. Am Heart J. (1969) 78(3):419–22. 10.1016/0002-8703(69)90048-9 5805987

[3] Khan H Hussain A Chaubey S Sameh M Salter I Deshpande R Acute aortic dissection type A: impact of aortic specialists on short and long term outcomes. J Card Surg. (2021) 36(3):952–8. 10.1111/jocs.15292 33415734

[4] Coady MA Rizzo JA Goldstein LJ Elefteriades JA. Natural history, pathogenesis, and etiology of thoracic aortic aneurysms and dissections. Cardiol Clin. (1999) 17(4):615–35. vii. 10.1016/S0733-8651(05)70105-3 10589336

[5] Trimarchi S Nienaber CA Rampoldi V Myrmel T Suzuki T Mehta RH Contemporary results of surgery in acute type A aortic dissection: the international registry of acute aortic dissection experience. J Thorac Cardiovasc Surg. (2005) 129(1):112–22. 10.1016/j.jtcvs.2004.09.005 15632832

[6] Chiappini B Schepens M Tan E Dell' Amore A Morshuis W Dossche K Early and late outcomes of acute type A aortic dissection: analysis of risk factors in 487 consecutive patients. Eur Heart J. (2005) 26(2):180–6. 10.1093/eurheartj/ehi024 15618075

[7] Harris KM Strauss CE Eagle KA Hirsch AT Isselbacher EM Tsai TT Correlates of delayed recognition and treatment of acute type A aortic dissection: the international registry of acute aortic dissection (IRAD). Circulation. (2011) 124(18):1911–8. 10.1161/CIRCULATIONAHA.110.006320 21969019

[8] Bashir M Shaw M Field M Kuduvalli M Harrington D Fok M Repair of type A dissection-benefits of dissection rota. Ann Cardiothorac Surg. (2016) 5(3):209–15. 10.21037/acs.2016.05.09 27386408 PMC4893525

[9] Zhang Y Chen T Chen Q Min H Nan J Guo Z. Development and evaluation of an early death risk prediction model after acute type A aortic dissection. Ann Transl Med. (2021) 9(18):1442. 10.21037/atm-21-4063 34733994 PMC8506734

[10] Zaschke L Habazettl H Thurau J Matschilles C Gohlich A Montagner M Acute type A aortic dissection: aortic dissection detection risk score in emergency care—surgical delay because of initial misdiagnosis. Eur Heart J Acute Cardiovasc Care. (2020) 9(3_suppl):S40–7. 10.1177/2048872620914931 32223297

[11] Sievers HH Rylski B Czerny M Baier ALM Kreibich M Siepe M Aortic dissection reconsidered: type, entry site, malperfusion classification adding clarity and enabling outcome prediction. Interact Cardiovasc Thorac Surg. (2020) 30(3):451–7. 10.1093/icvts/ivz281 31755925

[12] Khaladj N Shrestha M Peterss S Strueber M Karck M Pichlmaier M Ascending aortic cannulation in acute aortic dissection type A: the Hannover experience. Eur J Cardiothorac Surg. (2008) 34(4):792–6. disussion 796. 10.1016/j.ejcts.2008.05.014 18579405

[13] Shrestha M Martens A Behrendt S Maeding I Koigeldiyev N Haverich A. Is the branched graft technique better than the en bloc technique for total aortic arch replacement? Eur J Cardiothorac Surg. (2014) 45(1):181–6. discussion 186-7. 10.1093/ejcts/ezt357 23872460

[14] Martens A Koigeldiyev N Beckmann E Fleissner F Kaufeld T Krueger H. Do not leave the heart arrested. Non-cardioplegic continuous myocardial perfusion during complex aortic arch repair improves cardiac outcome. Eur J Cardiothorac Surg. (2016) 49(1):141–8. 10.1093/ejcts/ezv009 25669649

[15] Karck M Chavan A Hagl C Friedrich H Galanski M Haverich A. The frozen elephant trunk technique: a new treatment for thoracic aortic aneurysms. J Thorac Cardiovasc Surg. (2003) 125(6):1550–3. 10.1016/S0022-5223(03)00045-X 12830086

[16] Karck M Chavan A Khaladj N Friedrich H Hagl C Haverich A. The frozen elephant trunk technique for the treatment of extensive thoracic aortic aneurysms: operative results and follow-up. Eur J Cardiothorac Surg. (2005) 28(2):286–90. discussion 290. 10.1016/j.ejcts.2005.02.046 15922612

[17] Jakob H Tsagakis K Pacini D Di Bartolomeo R Mestres C Mohr F The international E-vita open registry: data sets of 274 patients. J Cardiovasc Surg (Torino). (2011) 52(5):717–23. . 21894139

[18] Shrestha M Pichlmaier M Martens A Hagl C Khaladj N Haverich A. Total aortic arch replacement with a novel four-branched frozen elephant trunk graft: first-in-man results. Eur J Cardiothorac Surg. (2013) 43(2):406–10. 10.1093/ejcts/ezs296 22653445

[19] Shrestha M Martens A Kaufeld T Beckmann E Bertele S Krueger H Single-centre experience with the frozen elephant trunk technique in 251 patients over 15 years. Eur J Cardiothorac Surg. (2017) 52(5):858–66. 10.1093/ejcts/ezx218 28977379

[20] Hagan PG Nienaber CA Isselbacher EM Bruckman D Karavite DJ Russman PL The international registry of acute aortic dissection (IRAD): new insights into an old disease. JAMA. (2000) 283(7):897–903. 10.1001/jama.283.7.897 10685714

[21] Fann JI Smith JA Miller DC Mitchell RS Moore KA Grunkemeier G Surgical management of aortic dissection during a 30-year period. Circulation. (1995) 92(9 Suppl):II113–21. 10.1161/01.CIR.92.9.113 7586393

[22] Lloyd-Jones DM. Cardiovascular health and protection against CVD: more than the sum of the parts? Circulation. (2014) 130(19):1671–3. 10.1161/CIRCULATIONAHA.114.012869 25273999

[23] Freundt M Kolat P Friedrich C Salem M Gruenewald M Elke G Preoperative predictors of adverse clinical outcome in emergent repair of acute type A aortic dissection in 15 year follow up. J Clin Med. (2021) 10(22):5370–80. 10.3390/jcm10225370 34830651 PMC8625674

[24] Hata M Shiono M Inoue T Sezai A Niino T Funahashi M Preoperative cardiopulmonary resuscitation is the only predictor for operative mortality of type A acute aortic dissection: a recent 8-year experience. Ann Thorac Cardiovasc Surg. (2004) 10(2):101–5. . 15209552

[25] Wang Z Ge M Chen T Chen C Zong Q Lu L Independent risk factors and the long-term outcomes for postoperative continuous renal replacement treatment in patients who underwent emergency surgery for type a acute aortic dissection. J Cardiothorac Surg. (2020) 15(1):100. 10.1186/s13019-020-01153-8 32414388 PMC7226713

[26] Dimagli A Angelini GD. Time is aorta?": timeliness of surgical repair in type A aortic dissection. J Card Surg. (2022) 37(6):1661–3. 10.1111/jocs.16412 35340069 PMC9314949

[27] Crawford TC Beaulieu RJ Ehlert BA Ratchford EV Black JH 3rd. Malperfusion syndromes in aortic dissections. Vasc Med. (2016) 21(3):264–73. 10.1177/1358863X15625371 26858183 PMC4876056

[28] Goldberg JB Lansman SL Kai M Tang GHL Malekan R Spielvogel D. Malperfusion in type A dissection: consider reperfusion first. Semin Thorac Cardiovasc Surg. (2017) 29(2):181–5. 10.1053/j.semtcvs.2016.10.017 28823325

[29] Bonser RS Ranasinghe AM Loubani M Evans JD Thalji NM Bachet JE Evidence, lack of evidence, controversy, and debate in the provision and performance of the surgery of acute type A aortic dissection. J Am Coll Cardiol. (2011) 58(24):2455–74. 10.1016/j.jacc.2011.06.067 22133845

[30] Shetty V Shetty DP Rao PV Hosabettu PK Subramanian S Vikneswaran G Determinant of outcome in late presenting type A aortic dissection. J Card Surg. (2022) 37(6):1654–60. 10.1111/jocs.16401 35285553

[31] Pacini D Leone A Belotti LM Fortuna D Gabbieri D Zussa C Acute type A aortic dissection: significance of multiorgan malperfusion. Eur J Cardiothorac Surg. (2013) 43(4):820–6. 10.1093/ejcts/ezs500 23137559

